# Renal phenotypes correlate with genotypes in unrelated individuals with tuberous sclerosis complex in China

**DOI:** 10.1186/s13023-022-02443-1

**Published:** 2022-07-23

**Authors:** Cong Luo, Ye Zhang, Yu-shi Zhang, Ming-Xin Zhang, Jun Ning, Min-Feng Chen, Yuan Li, Lin Qi, Xiong-Bing Zu, Yang-Le Li, Yi Cai

**Affiliations:** 1 Department of Urology, Xiangya Hospital, Central South University, No. 87 Xiangya Road, Changsha City, 410008 Hunan Province People’s Republic of China; 2 National Clinical Research Center for Geriatric Disorders, Xiangya Hospital, Central South University, Changsha City, 410008 Hunan Province People’s Republic of China; 3 Department of Oncology, NHC Key Laboratory of Cancer Proteomics, Xiangya Hospital, Central South University, No. 87 Xiangya Road, Changsha City, 410008 Hunan Province People’s Republic of China; 4 Department of Urology, Peking Union Medical College Hospital, Chinese Academy of Medical Sciences and Peking Union Medical College, No. 1 Shuaifuyuan, Dongcheng District, Beijing, 100730 People’s Republic of China; 5 Department of Urology, The Affiliated Hospital of Qingdao University, No. 16 Jiangsu Road, Shinan District, Qingdao City, 266000 Shandong Province China

**Keywords:** Tuberous sclerosis complex, Genotype, Renal phenotype, *TSC1*, *TSC2*

## Abstract

**Purpose:**

To explore the relationship between the genotype and renal phenotype in a Chinese cohort and guide clinical decision-making for treating tuberous sclerosis complex (TSC).

**Materials and methods:**

We reviewed 173 patients with definite TSC at three centers in China from September 2014 to September 2020. All the patients underwent *TSC1* and *TSC2* genetic testing as well as renal phenotypic evaluation. All analyses were performed using the SPSS software, version 19.0, with a cut-off *P* value of 0.05 considered statistically significant.

**Results:**

We identified variants in 93% (161/173) cases, including 16% *TSC1* and 77% *TSC2* variants. Analysis of the relationship between the genotype and renal phenotype, revealed that those with *TSC2* variants were more likely to develop severe renal AML (> 4) (*P* = 0.044). In terms of treatment, *TSC2* variants were more likely to undergo nephrectomy/partial nephrectomy (*P* = 0.036) and receive mTOR medication such as everolimus (*P* < 0.001). However, there was no significant difference between the two groups in terms of their response to the everolimus treatment.

**Conclusion:**

Patients with *TSC2* variants exhibit more severe renal phenotypes, especially those associated with renal angiomyolipomas (AML), and they often require nephrectomy/partial nephrectomy or mTOR medication. Detection of the genotype is helpful in TSC management.

**Supplementary Information:**

The online version contains supplementary material available at 10.1186/s13023-022-02443-1.

## Introduction

As an autosomal dominant neurocutaneous syndrome, tuberous sclerosis complex (TSC) is characterized by hamartomas in multiple organ systems, with an incidence rate of ~ 1/6000 to 1/10,000 [1]. TSC is believed to develop from a pathogenic variant of *TSC1* or, more commonly, *TSC2*. Coding proteins of *TSC1* and *TSC2* can form a functional complex repressing upstream of the mammalian target of rapamycin (mTOR). Either *TSC1* or *TSC2* variants can inactivate the TSC1/TSC2 complex, which results in the hyperactivation of the mTOR pathway and promotes cell growth and proliferation, leading to the development of benign tumors or hamartomas in multiple organ systems, including the skin, brain, eyes, heart, and kidneys. Therefore, therapies targeting mTOR have contributed to encouraging results in the treatment of TSC [2].

The *TSC1* gene has 23 exons extending over approximately 55 kb of the genomic DNA on chromosome 9q34.3. The TSC1 mRNA is transcribed from exons 3 to 23, and its subsequent protein hamartin is translated from the 8.6 kb mRNA transcript and has an estimated molecular weight of 130 kDa. On the other hand, the *TSC2* gene has 42 exons extending over approximately 40 kb of the genomic DNA on chromosome 16p13.3. Its subsequent protein tuberin is translated from the 5.5 kb mRNA transcript and has an estimated molecular weight of 200 kDa [3, 4]. The encoded proteins can form a heterotrimeric complex, termed the TSC protein complex, with TBC1 domain family member 7 (TBC1D7). Importantly, *TSC2* contains a highly conserved GTPase-activating protein (GAP) domain that enables the TSC protein complex to inhibit mTOR pathway by RAS homologue enriched in brain (Rheb) [5].

The renal phenotypes of TSC include renal AML, cysts, impaired kidney function and, rarely, renal cell carcinoma (RCC). Specifically, AML, a type of benign tumor, develops in the kidneys in up to 80% of TSC patients [6]. It contributes to chronic kidney disease (CKD) and intrarenal hemorrhage, thus acting as the most common cause of TSC-related mortality. Furthermore, cysts develop in approximately 30–45% of TSC patients and may be associated with acute or chronic kidney failure and resistant hypertension. RCC, affecting 2–3% of TSC patients, may be misdiagnosed as fat-poor AML [6]. These complications substantially worsen the patient survival rate and underscore the necessity for early diagnosis and prompt treatment of TSC. Thus, there is a crucial need for more optimized diagnostic protocols to assist medical practitioners in the early diagnosis of TSC and the evaluation of multiple organs to improve patient outcomes.

The correlation between TSC and neurological abnormalities has been widely documented [7, 8], but many TSC patients often seek urological treatment primarily because of the diagnosis of renal AML. In this study, we focused on the correlation between TSC genotypes and renal phenotypes that were not well discussed in large cohort studies performed previously. Intending to guide clinical therapy with precision for TSC patients, we further explored whether different genotypes affect mTOR inhibitor treatment response.

## Methods

### Study group

A cohort of all 173 unrelated patients with a definite diagnosis of TSC who had been treated at Xiangya Hospital Central South University, Peking Union Medical College Hospital and The Affiliated Hospital of Qingdao University from September 2014 to September 2020 was included in this study. The study was conducted in accordance with the declaration of Helsinki and local regulations, and obtained ethical approval. Definite diagnosis was defined as fulfilling 2 major criteria or 1 major criterion and ≥ 2 minor criteria recommended by the 2012 International Tuberous Sclerosis Complex Consensus Conference [9]. All patients underwent renal, pulmonary, and brain imaging evaluations as well as renal function tests, and some also underwent pulmonary function tests (n = 157), cardiotocography (n = 155), and pathological diagnosis (n = 44). Patients with at least one AML (diameter ≥ 30 mm) were treated with oral everolimus 10 mg per day for at least 3 months. Then they received follow-up radiographic evaluation at 3 and 6 months. The primary efficacy endpoint was achieving a ≥ 25% reduction in the total volume of target AML compared to baseline in patients with confirmed AML response.

### Renal phenotypic assessment

Patients were further categorized based on the renal angiomyolipoma staging criteria described previously [10]. The AML volumes were calculated based on the results of kidney computed tomography (CT) (n = 92) or magnetic resonance imaging (MRI) (n = 81). Moreover, the CKD stage (1–5) was determined by determining the glomerular filtration rate (GFR) that was calculated based on serum creatinine levels using the Chronic Kidney Disease Epidemiology (CKD-EPI) formula. Specifically, patients without renal angiomyolipoma were categorized as stage 0. On the other hand, if the AML stage could not be determined until the end of follow-up, these patients were also categorized as stage 0. Patients receiving hemodialysis were categorized as CKD stage 5 regardless of GFR values.

### Mutational analysis

Peripheral blood (10 mL) samples were collected from each patient for *TSC1*/*TSC2* gene detection. The mutational analysis was performed by the next-generation sequencing (NGS) at the Beijing Genomics Institute (Shenzhen, P.R. China) or the NHC Key Laboratory of Cancer Proteomics (Hunan Province, P.R. China). And all variants were verified by PCR-SSCP. Finally, they were compared with the LOVD databases (www.lovd.nl/TSC1; www.lovd.nl/TSC2) and classified into five categories according to the ACMG guideline [11]: class 5 (pathogenic), class 4 (likely pathogenic), class 3 (variant of unknown significance), class 2 (likely benign), and class 1 (benign).The possible effect of the newly identified mutations due to amino acid substitutions on protein function was determined using the online tools SIFT and PolyPhen2. Patients with predicted disease-associated mutations in the *TSC1* and *TSC2* genes were labeled accordingly. Patients with no definite findings or only intron mutations that unlikely to be pathogenic were labeled as “no mutation identified” (NMI) (Additional file 1).

### Statistical analysis

Continuous variables were reported as mean ± standard deviation (M ± SD) and categorical variables were reported as frequency counts and percentages (%). All statistical analyses were performed using the SPSS software, version 19.0 (SPSS, Chicago, IL, USA). Continuous variables were compared by Mann–Whitney U test, while categorical variables were compared by Chi-square test or Fisher's exact test, as appropriate. All the reported *P* values were 2-sided. A *P* value of < 0.05 was considered statistically significant, which was bold in the tables.

## Results

### The mutation rate of TSC1/TSC2 is 85%

**Fig. 1:**
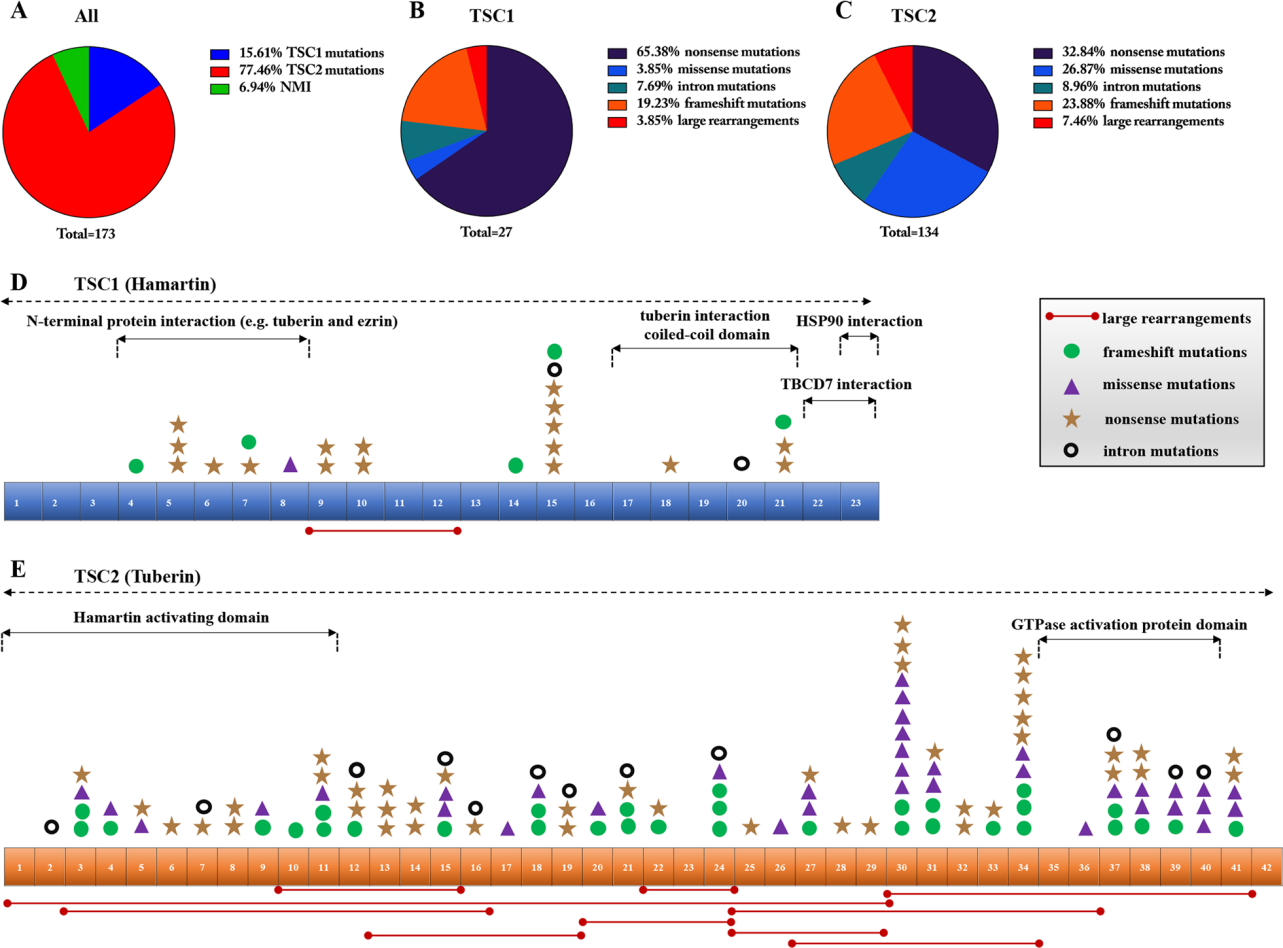
*TSC1* and *TSC2* gene mutation spectrum in Chinese patients. **A** The proportion of *TSC1*, *TSC2* gene and NMI in the cohort; **B** the mutation types of *TSC1*; **C** the mutation types of *TSC2*; **D** the distribution of various mutation types in *TSC1*; **E** the distribution of various mutation types in *TSC2*

In general, the mutation rate of *TSC1*/*TSC2* was 93% among the 173 patients. Specifically, we identified 27 (16%) variants in *TSC1*, 134 (77%) in *TSC2* and 12 (7%) NMI (Fig. 1A). Moreover, the types of *TSC1* and *TSC2* variants are summarized in Fig. 1B–C. *TSC1* variants mainly included 65% nonsense and 19% frameshift events, whereas the *TSC2* variants mainly included 33% nonsense, 27% missense and 24% frameshift events. The distributions of different variants within the *TSC1* and *TSC2* genes are shown in Fig. 1D–E. Furthermore, the most common mutation site in the *TSC1* gene was located in exon 15 (7/27; 26% of total *TSC1* variants), whereas that in the *TSC2* gene was located in exon 30 (15/134; 11% of total *TSC2* variants). Exons 35–39 of the *TSC2* gene are responsible for encoding the GTPase activating-protein (GAP) domain, which is essential for tuberin function. We identified 18 variants within these five exons that accounted for almost 13% of all the *TSC2* variants identified. They included all four mutation types (nonsense mutations, missense mutations, frameshift mutations, and large rearrangements). Next, we performed NGS on these patients, as well as analyzed the data using the LOVD database (http://www.LOVD.nl/TSC1, http://www.LOVD.nl/TSC2) and related studies. The majority of the variants had been reported previously, most of which were pathogenic or likely pathogenic variants, and only 5 variants (1 *TSC1* and 4 *TSC2*) were benign or likely benign. In addition, 14 novel variants were found of uncertain significance, including 3 *TSC1* variants and 11 *TSC2* variants (Table 1). These NMI, intron mutations, variants of uncertain significance, and benign/likely benign variants were excluded from the subsequent analysis. We mainly focused on the correlation between pathogenic/likely pathogenic variants with the renal phenotypes.

**Table 1:** New variants detected by next-generation sequencing

No	Sex	Age	Mutant	Nucleotide change	Protein change	Mutation type
1	Female	16	*TSC1*	c. 605_606insA	Phe202Leufs*16	Frameshift mutations
2	Female	57	*TSC1*	c.1018G > T	Glu340*	Nonsense mutations
3	Female	24	*TSC1*	c.361A > T	Lys121*	Nonsense mutations
4	Female	36	*TSC2*	c.3683_3684 insG	Leu1228Leufs*6	Frameshift mutations
5	Female	36	*TSC2*	c.788_789 insC	Leu263Leufs*75	Frameshift mutations
6	Male	30	*TSC2*	c.2738_2739 insT	Thr913Thrfs*2	Frameshift mutations
7	Male	31	*TSC2*	c.4006_4007 insC	Ser1336Serfs*78	Frameshift mutations
8	Female	30	*TSC2*	c.3601_3602 insGGCCC	Thr1203Glyfs*9	Frameshift mutations
9	Female	46	*TSC2*	c.203_204 insA	Ala68Alafs*7	Frameshift mutations
10	Male	16	*TSC2*	c.3271insTCCG	Gly1091Valfs*78	Frameshift mutations
11	Female	29	*TSC2*	c.1258G > T	Glu420*	Nonsense mutations
12	Female	50	*TSC2*	c.4972A > T	Lys1658*	Nonsense mutations
13	Female	24	*TSC2*	c.3838G > T	Gln1280*	Nonsense mutations
14	Female	23	*TSC2*	c.2319 delA	Leu773Leufs*56	Frameshift mutations

### Female or familial patients are more likely to carry TSC1 variants

The demographic characteristics and variant data of all the patients are summarized in Table 2. The age of the patients with *TSC1* variants was not significantly different from that of the patients with *TSC2* variants (*P* = 0.37). Female TSC patients were more likely to carry *TSC1* variants rather than *TSC2* variants, as 17 (17/21, 81%) *TSC1* variants were identified in female patients, whereas only 59 (59/107, 55%) *TSC2* variants were identified in female patients (*P* = 0.031). Moreover, 15 (15/21, 71%) *TSC1* variants and 97 (97/107, 91%) *TSC2* variants were detected as de novo variants, whereas familial inheritance was observed in the remaining variants. Overall, the proportion of de novo variants was significantly higher in *TSC2* variants than in *TSC1* variants (*P* = 0.015). Regarding the symptoms considered for determining the major or minor criteria of TSC, there was no significant difference in the incidence of different phenotypes among different genotype subgroups.

**Table 2:** Clinical characteristics by genotype in patients with tuberous sclerosis complex

	*TSC1*	*TSC2*	*P* value
	(n = 21)	(n = 107)	*TSC1* versus *TSC2*
Age (years), median (range)	30 (6–54)	31 (4–59)	0.37
Sex			**0.031**
Male	4	48	
Female	17	59	
Familial/de novo			**0.015**
Familial	6	10	
De novo	15	97	
Major criteria			
Angiofibromas (≥ 3) or forehead	14/21	88/107	0.10
Hypomelanotic macules (≥ 3)	15/21	85/107	0.42
Ungual fibromas (≥ 2)	7/21	57/107	0.095
Chagrin patch	10/21	61/107	0.42
Multiple retinal hamartomas	3/15	11/81	0.45
Cortical dysplasia	2/21	12/107	0.99
SEN	20/21	86/107	0.12
SEGA	1/21	4/104	0.99
Cardiac rhabdomyoma	3/19	5/102	0.11
LAM (women)	6/18	26/105	0.064
Renal AML (≥ 2)	20/21	103/107	0.99
Minor criteria			
Confetti skin lesions	3/21	19/107	0.99
Dental enamel pits (≥ 3)	5/21	15/106	0.27
Intraoral fibromas (≥ 2)	2/21	12/106	0.99
Retinal achromatic patch	0/14	0/76	–
Non-renal hamartomas	5/20	22/105	0.69
Multiple renal cysts	1/20	12/106	0.69

*SEN* subependymal nodules, *SEGA* subependymal giant cell astrocytoma, *LAM* lymphagioleiomatosis, *AML* angiomyolipomas

### Patients with TSC2 variants are more likely to develop more severe renal complications

To determine the correlation between renal phenotypes and genotypes, we compared the renal phenotypes of *TSC1* variants to those of *TSC2* variants (Table 3).we found that patients with *TSC2* variants were more likely to develop more severe versions of renal AML (> 4) (*P* = 0.044). Significant differences with regard to the treatment received for renal AML were observed between those groups. *TSC2* variants were more likely to receive nephrectomy/partial nephrectomy (*P* = 0.036). And they preferred to receive mTOR medication such as everolimus (*P* < 0.001). For these patients carrying with pathogenic mutations, a total of 37 patients underwent pathological diagnosis, including 27 AML (1 *TSC1* and 26 *TSC2*), 6 epithelioid AML (6 *TSC2*) and 4 RCC (1 *TSC1* and 3 *TSC2*). There was no significant difference of endogenous creatinine clearance rate (Ccr) and CKD stage among the different genotype subgroups. Moreover, there were 3 patients with *TSC2* variants who had polycystic kidney disease (PKD). The four patients with AML and RCC were mainly between 40 and 45 years old. All of them had neurological involvement, and multiple subependymal nodules were found on cranial MRI. Three of them received everolimus treatment but none had a good response.

**Table 3:** Renal complications by genotype in patients with tuberous sclerosis complex

	*TSC1*	*TSC2*	*P* value
	(n = 21)	(n = 107)	*TSC1* versus *TSC2*
Renal AML			
AML	20/21	104/107	0.52
Bilateral AML	19/21	103/107	0.26
Diameter of the largest AML (cm)	4.2 ± 1.7	5.6 ± 4.0	0.11
Volume of largest AML (cm^3^)	34 ± 39	159 ± 440	0.20
AML stage			
≤ 4	18	66	**0.044**
> 4	3	41	
Surgery/invasive procedure	7/21	54/107	0.15
Renal embolization	6/21	31/107	0.97
Nephrectomy/partial	2/21	35/107	**0.036**
mTOR Medication (Everolimus)	4/21	68/107	**< 0.001**
Renal cysts			0.69
Yes	1	12	
No	20	94	
Renal cell carcinomas			0.52
Yes	1	3	
No	20	104	
Ccr (ml/min)	95 ± 20	90 ± 31	0.54
CKD stage			0.073
≤ 2	20	82	
> 2	1	25	
PKD			0.99
Yes	0	3	
No	21	104	

*AML* angiomyolipomas, *Ccr* endogenous creatinine clearance rate, *CKD* chronic kidney disease, *PKD* polycystic kidney disease

### Genotype does not affect the response of AML volume to mTOR medication

We investigated the effect of genomic factors on the reduction in AML volume after mTOR treatment (Table 4). A total of 72 patients received everolimus for 3 months, including 68 patients with *TSC2* variants and 4 patients with *TSC1* variants. A total of 70 patients received everolimus for 6 months, including 66 patients with *TSC2* variants and 4 patients with *TSC1* variants. Before receiving the medication, the volume of target AML in patients with *TSC2* variants was 70 ± 76 cm^3^, whereas that in patients with *TSC1* variants or NMI was 235 ± 538 cm^3^. There was no significant difference in AML volume between the two groups (*P* = 0.54). In general, 76% of patients with *TSC2* variants and 75% of patients with *TSC1* variants responded to the medication at 3 months (*P* = 0.99), while 85% of patients with *TSC2* variants and 100% of patients with *TSC1* variants responded to the medication at 6 months (*P* = 0.99). At the 6-month follow-up, one *TSC2* variant stopped receiving everolimu due to adverse event, and another *TSC2* variant withdrew from the cohort for economic reason. There was no significant difference in age, gender and family history between two groups. Moreover, there was no statistical difference in the reduction of AML volume after the everolimus treatment and the response rate between two groups.

**Table 4:** Response of angiomyolipoma volume to everolimus by genotype

	*TSC1*	*TSC2*	*P* value
	(n = 4)	(n = 68)	*TSC1* versus *TSC2*
AML volume at baseline (mean ± SD, cm^3^)	70 ± 76	235 ± 538	0.54
Age range (years)	29–45	16–59	0.079
Median age (years)	36	34	
Sex			0.63
Male	2	25	
Female	2	43	
Familial/de novo			0.17
Familial	2	12	
De novo	2	56	
Everolimus (3 months)			
No. of response (%)	3 (75)	52 (76)	0.99
% reduction from baseline value (mean ± SD, %)	39 ± 14	38 ± 17	0.89
Everolimus (6 months)			
No. of response (%)	4 (100)	56 (85)	0.99
% reduction from baseline value (mean ± SD, %)	41 ± 15	43 ± 18	0.87

*AML* angiomyolipomas

## Discussion

In this study, we identified 161 variants in 173 unrelated patients (93%), including 27 (16%) with *TSC1* variants and 134 (77%) with *TSC2* variants, which is higher than that reported in other countries [12–15]. This is due to the NGS applied in this study. Several intron variants of *TSC1*/*TSC2* have been found in our study. Because they are usually not pathogenic or likely pathogenic, these variants have been excluded from subsequent phenotype related analysis. Moreover, we identified 14 novel *TSC1*/*TSC2* variants, which expands the public database and calls for researchers to investigate its functions. Mosaicism may partially explain the 12 NMI case, which has been found in approximately 1/2 NMI patients [16]. Further locus heterogeneity in TSC also remains a theoretical possibility. In the present study, we analyzed the mutation spectrum of *TSC1*/*TSC2* genes based on a Chinese TSC cohort with large sample size. Multiple types of variants were detected, including frameshift, nonsense, missense, and large rearrangement mutations. Further stratification analysis showed that nonsense mutations were the predominated mutation type across *TSC1* variants, whereas frameshift and missense mutations were more common in *TSC2* variants, which is consistent with the results of another Chinese cohort study [17]. Moreover, in this study, we identified that exon 15 is the most frequent mutation site in *TSC1* variants as reported previously [17]. However, exon 30 was uniquely identified as the most frequent mutation site in *TSC2* variants in our study, which may be a hotspot mutation region in Chinese patients with TSC since previous studies have rarely reported this mutation site. Furthermore, previous studies have found *TSC2* variant sites scattered throughout the gene but more common in exons 35–39 [18]. They encode the GAP domain, which affects mTOR inhibition by the TSC protein complex. Therefore, variants within this region play an important role in the pathogenesis of TSC and correlate with poor clinical outcomes [14]. We also observed several *TSC2* variants within this region in our study. By performing genomic analysis of TSC patients, the mutation spectrum and distribution profiles of the two essential genes (*TSC1* and *TSC2*) in the Chinese TSC population have gradually become comprehensive and clear. Intriguingly, 14 novel variants of *TSC1*/*TSC2* genes were confirmed in our study, which can be used as a reliable reference for future genetic testing of TSC patients.

We further identified a significant correlation between the clinical characteristics and genotypes of patients with TSC. There was no significant difference in age distribution among the two groups in the study. However, it has been suggested that the onset age of renal AML among *TSC2* variants was earlier, which requires further exploration [19]. The role of gender in TSC-associated pathogenesis has been studied previously in both animal models and human studies but the findings remain controversial. A previous study found that males with TSC have a greater risk of learning disorders and autism than females with TSC [20]. However, a large sample cohort study in the United States suggested that *TSC1*/*TSC2* variants showed no significant distributive difference between male and female patients [15]. In our cohort, a contrary conclusion was drawn that males with TSC were more likely to carry *TSC2* variants. Further research by including independent cohorts with larger sample sizes is needed to verify whether sex hormones are involved in the *TSC1*/*TSC2* mutations and TSC pathogenesis. Additionally, we found that 29% *TSC1* variants were familial variants, whereas only 9% *TSC2* variants were inherited from families, which is consistent with the results of the previous studies [15, 21, 22]. We confirmed the higher frequency of familial *TSC1* variants compared to that of familial *TSC2* variants, which is attributed to the smaller size and simpler structure of the *TSC1* genomic locus, and the rarity of missense mutations, frames shift mutations and large rearrangements at the locus [12]. A large number of *TSC1* variants was observed in familial cases probably because patients with *TSC1* variants are less likely to be affected by severe neurocognitive impairment and are therefore more likely to have offspring [13, 23].

Some researchers have attempted to determine genotype/phenotype correlations with TSC but could not conclusively establish a relationship between these factors [24, 25]. A robust correlation has been previously established between *TSC2* variants and poor clinical phenotypes including skin, neurological, and renal features [15, 26]. A previous study failed to found positive results between renal AML and genotypes in Chinese population [27]. While another Chinese cohort study found that the renal AML volume of *TSC2* variants was significantly larger than that of *TSC1* variants [28]. More recently, the correlation between mutational sites and renal AML have been evaluated [29]. It identified some mutational sites, such as *TSC2* mutations in the transcription activation domain 1 coding genes, had higher risk of renal AML. However, only the number of patients with or without renal AML were demonstrated. The specific details about renal AML were not analyzed. Compared with these study, our strength is that this is a multi-center study with relatively large sample size. More importantly, we also focused on treatment options as well as other TSC-associated kidney diseases, such as renal cysts, PKD, etc. in addition to comparisons of renal AML. Specifically, we found that patients with *TSC2* variants were more likely to develop more advanced AML (AML stage > 4). Besides, these *TSC2* variants often received nephrectomy/partial nephrectomy or mTOR medication such as everolimus. This reflects that *TSC2* variants may be associated with more severe renal phenotypes. And the history of nephrectomy/partial nephrectomy may contribute to some of the negative findings. Previous studies have reported that *TSC2* variants were more likely to develop more severe CKD [22, 27]. Although there were no difference between the two groups in our study, we can see that there was a trend. Overall, *TSC2* variants are more likely to have more severe renal phenotype. Thus, in the clinical setting, patients with *TSC2* variants should receive a more comprehensive renal evaluation and be treated promptly if renal abnormalities are detected. There were 4 patients (4/173, 2%) accompanied with RCC, which is consistent with the incidence reported in previous article [30]. It has been defined as RCC with fibrous stroma (RCC-FMS) (formerly RCC with leiomyomatous or smooth muscle stroma) by the The Genitourinary Pathology Society (GUPS) [31]. Among these 4 patients, 3 were treated with everolimus but did not achieved satisfactory outcomes. Therefore, it remains to be explored whether mTOR inhibitors play a role in TSC accompanied with RCC. There were 3 patients with *TSC2* muations suffered PKD in this study, with the mutation type of large rearrangements. Because *TSC2* gene is adjacent to *PKD1* gene, large deletion involving these two genes may lead to *PKD1/TSC2* continuous gene deletion syndrome (CGS). Because we mainly focused on the mutations of *TSC1* and *TSC2*, *PKD1* mutations were not detected. The latest research suggested CGS screening for patients with PKD and TSC-associated renal neoplasia as well as TSC patients with cystic renal disease [30]. This is worthy of further study in the follow-up.

Given that TSC results from the dysregulation of the mTOR signaling pathway, the advent of mTOR inhibitors such as everolimus, an analog of rapamycin, has provided great therapeutic promise in treating TSC [32]. It has been reported that the most significant reduction in renal AML growth by everolimus usually occurred within the initial 3–6 months of treatment, whereafter the tumor volume stabilized or gradually decreased. Therefore, we mainly followed up their renal AMLs at 3 and 6 months after everolimus treatment. We previously confirmed that everolimus was well tolerated and showed promising efficacy in Chinese patients with TSC-RAML [33]. In this study, the overall response rate to everolimus treatment was approximately 86% at 6 months, which was even higher than the previous report [34]. This further confirmed the effectiveness of everolimus in the treatment of renal angiomyolipoma. For these non-responders, most of them suffered renal AML with large volume, which is consistent with our previous study [35]. Further we compared the response of patients with different genotypes to the everolimus treatment. There was no statistical difference in the reduction of AML volume and the response rate between the two groups after receiving the medication. Therefore, studies using larger patient populations should be performed to determine whether the efficacy of everolimus is related to the genotype.

There were some limitations in this study. First, our study lacks precise and concrete data type since we defined the renal phenotype as the primary outcome, which is a qualitative data type rather than a quantitative one. The relationship between more quantitative renal phenotype and genotype needs to be assessed to help clinicians in comprehensively evaluating TSC patients and further guide precision medicine at the clinical stage. Second, the sample size of our cohort was not large enough, especially the number of patients treated with everolimus. Thus, our results is insufficiently powered to compare the impact of *TSC1* versus *TSC2* genotype on the response of AML volume to mTOR medication. Future studies should include more independent cohorts with larger patient populations to investigate the underlying relationships between genotypes and renal phenotypes as well as treatment response in TSC patients. Moreover, because of technical limitations, we were unable to measure the blood concentration. So we controlled the dose to reduce the bias.

## Conclusion

In summary, the overall positive *TSC1*/*TSC2* mutation detection rate in patients with TSC was 85% in our study. Those with *TSC2* variants were associated with more severe renal phenotypes compared with those with *TSC1* variants. Detection of the genotype is helpful in TSC management, which can be helpful for determining risk profiles, and in turn guide medical practitioners to treat TSC patients in a more precise manner with the hope to prevent complications and improve therapeutic outcomes.

## Supplementary Information

**Additional file 1.** Summary of mutational analysis.

## Data Availability

Please contact author (Yi Cai, cai-yi@csu.edu.cn) for data requests.

## References

[1] Osborne JP, Fryer A, Webb D (1991). Epidemiology of tuberous sclerosis. Ann N Y Acad Sci.

[2] Crino PB, Nathanson KL, Henske EP (2006). The tuberous sclerosis complex. N Engl J Med.

[3] Xu L, Sterner C, Maheshwar MM, Wilson PJ, Nellist M, Short PM (1995). Alternative splicing of the tuberous sclerosis 2 (TSC2) gene in human and mouse tissues. Genomics.

[4] European Chromosome 16 Tuberous Sclerosis Consortium (1993). Identification and characterization of the tuberous sclerosis gene on chromosome 16. Cell.

[5] Zhang Y, Gao X, Saucedo LJ, Ru B, Edgar BA, Pan D (2003). Rheb is a direct target of the tuberous sclerosis tumour suppressor proteins. Nat Cell Biol.

[6] Henske EP, Jóźwiak S, Kingswood JC, Sampson JR, Thiele EA (2016). Tuberous sclerosis complex. Nat Rev Dis Primers.

[7] Curatolo P, Moavero R, Roberto D, Graziola F (2015). Genotype/phenotype correlations in tuberous sclerosis complex. Semin Pediatr Neurol.

[8] Chu-Shore CJ, Major P, Camposano S, Muzykewicz D, Thiele EA (2010). The natural history of epilepsy in tuberous sclerosis complex. Epilepsia.

[9] Northrup H, Krueger DA (2013). Tuberous sclerosis complex diagnostic criteria update: recommendations of the 2012 Iinternational Tuberous Sclerosis Complex Consensus Conference. Pediatr Neurol.

[10] Eijkemans MJ, van der Wal W, Reijnders LJ, Roes KC, van Waalwijk van Doorn-Khosrovani SB, Pelletier C (2015). Long-term follow-up assessing renal angiomyolipoma treatment patterns, morbidity, and mortality: an observational study in tuberous sclerosis complex patients in the Netherlands. Am J Kidney Dis.

[11] Richards S, Aziz N, Bale S, Bick D, Das S, Gastier-Foster J (2015). Standards and guidelines for the interpretation of sequence variants: a joint consensus recommendation of the American College of Medical Genetics and Genomics and the Association for Molecular Pathology. Genet Med.

[12] Rosengren T, Nanhoe S, de Almeida LGD, Schönewolf-Greulich B, Larsen LJ, Hey CAB (2020). Mutational analysis of TSC1 and TSC2 in Danish patients with tuberous sclerosis complex. Sci Rep.

[13] Niida Y, Wakisaka A, Tsuji T, Yamada H, Kuroda M, Mitani Y (2013). Mutational analysis of TSC1 and TSC2 in Japanese patients with tuberous sclerosis complex revealed higher incidence of TSC1 patients than previously reported. J Hum Genet.

[14] Choi JE, Chae JH, Hwang YS, Kim KJ (2006). Mutational analysis of TSC1 and TSC2 in Korean patients with tuberous sclerosis complex. Brain Dev.

[15] Au KS, Williams AT, Roach ES, Batchelor L, Sparagana SP, Delgado MR (2007). Genotype/phenotype correlation in 325 individuals referred for a diagnosis of tuberous sclerosis complex in the United States. Genet Med.

[16] Tyburczy ME, Dies KA, Glass J, Camposano S, Chekaluk Y, Thorner AR (2015). Mosaic and intronic mutations in TSC1/TSC2 explain the majority of TSC patients with no mutation identified by conventional testing. PLoS Genet.

[17] Jiangyi W, Gang G, Guohai S, Dingwei Y (2020). Germline mutation of TSC1 or TSC2 gene in Chinese patients with bilateral renal angiomyolipomas and mutation spectrum of Chinese TSC patients. Aging (Albany NY).

[18] Salussolia CL, Klonowska K, Kwiatkowski DJ, Sahin M (2019). Genetic etiologies, diagnosis, and treatment of tuberous sclerosis complex. Annu Rev Genomics Hum Genet.

[19] Kingswood JC, Belousova E, Benedik MP, Carter T, Cottin V, Curatolo P (2019). Renal angiomyolipoma in patients with tuberous sclerosis complex: findings from the TuberOus SClerosis registry to increase disease Awareness. Nephrol Dial Transplant.

[20] Smalley SL, Tanguay PE, Smith M, Gutierrez G (1992). Autism and tuberous sclerosis. J Autism Dev Disord.

[21] Povey S, Burley MW, Attwood J, Benham F, Hunt D, Jeremiah SJ (1994). Two loci for tuberous sclerosis: one on 9q34 and one on 16p13. Ann Hum Genet.

[22] Sancak O, Nellist M, Goedbloed M, Elfferich P, Wouters C, Maat-Kievit A (2005). Mutational analysis of the TSC1 and TSC2 genes in a diagnostic setting: genotype–phenotype correlations and comparison of diagnostic DNA techniques in Tuberous Sclerosis Complex. Eur J Hum Genet.

[23] van Eeghen AM, Black ME, Pulsifer MB, Kwiatkowski DJ, Thiele EA (2012). Genotype and cognitive phenotype of patients with tuberous sclerosis complex. Eur J Hum Genet.

[24] Niida Y, Lawrence-Smith N, Banwell A, Hammer E, Lewis J, Beauchamp RL (1999). Analysis of both TSC1 and TSC2 for germline mutations in 126 unrelated patients with tuberous sclerosis. Hum Mutat.

[25] van Slegtenhorst M, Verhoef S, Tempelaars A, Bakker L, Wang Q, Wessels M (1999). Mutational spectrum of the TSC1 gene in a cohort of 225 tuberous sclerosis complex patients: no evidence for genotype–phenotype correlation. J Med Genet.

[26] Dabora SL, Jozwiak S, Franz DN, Roberts PS, Nieto A, Chung J (2001). Mutational analysis in a cohort of 224 tuberous sclerosis patients indicates increased severity of TSC2, compared with TSC1, disease in multiple organs. Am J Hum Genet.

[27] Li S, Zhang Y, Wang Z, Yang Y, Gao W, Li D (2018). Genotype–phenotype correlation of patients with tuberous sclerosis complex-associated renal angiomyolipoma: a descriptive study. Hum Pathol.

[28] Ni J, Yan F, Qin W, Yu L, Zhang G, Liu F (2019). Mutational analysis of renal angiomyolipoma associated with tuberous sclerosis complex and the outcome of short-term everolimus therapy. Sci Rep.

[29] Zhang N, Wang X, Tang Z, Qiu X, Guo Z, Huang D (2020). The correlation between tuberous sclerosis complex genotype and renal angiomyolipoma phenotype. Front Genet.

[30] Gupta S, Lohse CM, Rowsey R, McCarthy MR, Shen W, Herrera-Hernandez L (2022). Renal neoplasia in polycystic kidney disease: an assessment of tuberous sclerosis complex-associated renal neoplasia and PKD1/TSC2 contiguous gene deletion syndrome. Eur Urol.

[31] Trpkov K, Williamson SR, Gill AJ, Adeniran AJ, Agaimy A, Alaghehbandan R (2021). Novel, emerging and provisional renal entities: the Genitourinary Pathology Society (GUPS) update on renal neoplasia. Mod Pathol.

[32] Peron A, Au KS, Northrup H (2018). Genetics, genomics, and genotype–phenotype correlations of TSC: insights for clinical practice. Am J Med Genet C Semin Med Genet.

[33] Cai Y, Guo H, Wang W, Li H, Sun H, Shi B (2018). Assessing the outcomes of everolimus on renal angiomyolipoma associated with tuberous sclerosis complex in China: a 2 years trial. Orphanet J Rare Dis.

[34] Geynisman DM, Kadow BT, Shuch BM, Boorjian SA, Matin SF, Rampersaud E (2020). Sporadic angiomyolipomas growth kinetics while on everolimus: results of a phase II trial. J Urol.

[35] Wang W, Guo H, Shi B, Sun H, Li H, Zhang Y (2019). CT characteristics predict the response to everolimus or sirolimus of renal angiomyolipomas in patients with tuberous sclerosis complex. Int Urol Nephrol.

